# The Park is Ruining our Livelihoods. We Support the Park! Unravelling the Paradox of Attitudes to Protected Areas

**DOI:** 10.1007/s10745-017-9941-2

**Published:** 2017-10-18

**Authors:** Adrian Martin, Rodd Myers, Neil M. Dawson

**Affiliations:** Faculty of Social Science, School of International Development, Global Environmental Justice Group, University of East Anglia, Norwich, NR4 7TJ UK

**Keywords:** Conservation, Parks, Nam Et-Phou Louey, Lao PDR, Environmental justice

## Abstract

Despite considerable field-based innovation and academic scrutiny, the nexus between conservation approaches, local support for parks and park effectiveness remains quite puzzling. Common approaches to understanding notions of environmental justice are to understand distributional and procedural issues, representation in decision making, and recognition of authorities and claims. We took a different approach and analysed environmental justice claims through institutional, ideational and psychological lenses. We sought to understand how the national park could have such broad support from local communities despite their acknowledgement that it severely curtailed their livelihoods. We conducted 100 household interviews in three villages that border Nam Et-Phou Louey National Protected Area. Our study found that villagers 1) hold on to broken promises by the State for agricultural activities and alternative revenues without fully changing forest use behaviours; 2) were influenced heavily by the ‘educational’ programmes by the State; 3) accepted the authority of the State and lack of participation in decision-making based on historical experiences and values; 4) justified their burdens by over-emphasising the positive aspects of the park. Our findings present a complementary framework to explain environmental justice claims, allowing for a nuanced analysis of how people respond to justices and injustices, and specifically how injustices can be identified through proven social science concepts.

## Introduction

It is widely assumed that protected areas (PAs) will be more effective where they are well supported by local residents. To garner this support, various forms of community-based conservation became popular in the 1980s, based on the logic that people would be on the side of conservation if they were more involved in decision-making and if protected areas delivered tangible economic benefits (Hutton *et al*. 2005). By the late 1990s there was rising scepticism about this logic because researchers found little evidence that people friendly approaches were leading to the expected improvement in conservation effectiveness (Wells *et al*. 1999; Salafsky and Wollenberg 2000; Ferraro 2001; Brockington *et al*. 2008). Indeed, some were so disenchanted with community-based approaches that they called for a return to more protectionist approaches to conservation (Oates 1995; Terborgh 1999). More recently, the tide has turned again, with much stronger evidence emerging, showing that community participation and livelihood benefits are indeed more likely to contribute to conservation effectiveness than conservation without local participation (Ostrom and Nagendra 2006; Chhatre and Agrawal 2009; Persha *et al*. 2010; Persha *et al*. 2011; Coleman and Fleischman 2012; Oldekop *et al.* 2015; Cinner *et al*. 2016). The assumption that community support contributes to effective conservation appears to be standing the test of time.

However, whilst evidence for the positive effects of people friendly conservation approaches has strengthened, we still have limited understanding of what makes people support conservation. There are cases that appear to defy conventional logic, in which local collective action in support of conservation occurs despite a lack of any significant livelihood benefits. Around the Bwindi National Park in Uganda, for example, major improvements in support for the park, evidenced by significant reductions in conflict, are often attributed to the range of income generating projects and park revenue sharing schemes introduced since the late 1990s (Blomley 2010; Baker *et al*. 2012; Sandbrook 2010). Yet the costs of living near the park continue to be perceived by many to outweigh any benefits (Bush and Mwesigwa 2008; Martin *et al*. 2015), with one study finding that the park results in an average net loss of 12.5% of household income for adjacent populations (Tumusiime and Vedeld 2015).

It seems paradoxical that one would express support for a protected area that at best brings no benefits or at worst is a cause of hardship, yet this phenomenon might be quite commonplace. Newmark *et al*. (1993) studied support for five protected areas in Tanzania, finding that 71% of interview respondents were opposed to degazetting the parks, but that only 47% were able to specify anything good coming from the parks. Karanth and Nepal (2012) found that 81% of respondents in India and Nepal expressed positive attitudes towards neighbouring parks, despite high losses arising from living near them. Walpole and Goodwin (2001) found that 93.7% of their sample in Indonesia supported conservation but again that this was not linked to benefits arising from conservation. Similar observations of the complex and sometimes contradictory connections between general support for parks and perceived benefits have been observed in Myanmar (Allendorf *et al*. 2006), Nepal (Allendorf 2007) Benin (Vodouhê *et al*. 2010/9) and Ethiopia (Tessema *et al*. 2010).

This paper arises from our own difficulty in making sense of interview data obtained around the Nam Et-Phou Louey National Protected Area (NPA) in Northern Laos. As with the studies just cited, we found high levels of general support for the NPA, but at the same time found it hard to explain this via simple conventional logics related to economic benefits or to participation. Similarly to Allendorf (2007), we also suspected that individuals could hold apparently complex and contradictory sets of perceptions, displaying elements of support and opposition at the same time, rather than holding simple binary attitudes. High (2014) has also observed such apparently contradictory views in Laos, specifically in relations between rural communities and the State. Our aim then is to better understand the complexities around expressions of support for protected areas, especially with regard to how these link with perceptions of material benefits and governance.

So why would local people express support for a PA when they also identify it as a cause of their own economic hardship? In our attempt to explain this puzzle, we follow recent scholarship in assuming that support for parks does not flow from economic considerations alone, but from a range of factors that contribute to perceptions of legitimacy, fairness, and wellbeing. These factors include different categories of things to which people attach value and meaning, including distribution of harms and benefits, procedures for participation and decision-making and recognition of rights and identities (Schlosberg 2007; Martin *et al*. 2013; Pascual *et al*. 2014). However, whilst we take on board the multiple forms of value identified in this literature and their potential influence on expressions of support for a protected area, we adopt a different analytical approach. Instead of choosing types of value as our primary analytical category, we find it more helpful to employ different types of explanation for human behaviour, something that is often overlooked in socio-political studies (Daigneault and Béland 2015). More specifically, we seek to understand respondents’ views about the park through three different social scientific traditions of explaining behaviour: institutional, ideational and psychological.

i) *Institutional* forms of explanation seek to understand individual and group behaviour as shaped by their position in relation to external conditions, such as rules and governance structures (Parsons 2007). This external environment is predominantly constituted by social institutions, namely the formal and informal rules and norms that lead to recognisable patterns of behaviour (North 1990). Parsons (2007) imagines this as an obstacle course of constraints and incentives, with the observed regularities of behaviour providing the evidence that these institutions are indeed what are patterning human responses. In this analytical approach, humans are viewed as rational beings, responding in similar ways to shared ‘rules of the game’ and sharing a rational basis for social justice. For example, Lejano (2006) finds that an institutional analysis is important for understanding behavioural responses to peace parks; however, he finds that there is also a need to understand cultural and historical foundations of behaviour (what we call ideational explanations).
ii) *Ideational* explanations are concerned with particular, place-bound ways of thinking about and interpreting external conditions and are sometimes equated to cultural interpretations (c Orloff and Palier 2009). They are affected by the ways in which actors influence (or impose upon) one another and both shape and are shaped by institutions (Carstensen and Schmidt 2016). These subjective ways of thinking are both cognitive and affective in nature and formed individually and socially, often over historical time periods (Parsons 2007; Satyal 2011). They shape the ways in which actors understand their environment and contexts (Béland 2009). Given that different groups of people have different experiences, and have developed different cultures, there are in fact a plurality of rational responses to any one external signal (Stears 2003; He and Sikor 2015). In order to understand behaviour it is therefore not enough to understand the effect of institutions. One also needs to explore the back history shaping the identities of particular groups to understand why they respond to institutions in particular rather than general ways. For example, Sikor and Cầm (2016) explore local and state-level land governance histories to understand how a forest conservation project in Vietnam ended up selecting a rocky and treeless area to protect, whilst not being able to garner support to protect a genuine forested area.
iii) *Psychological* explanations are distinguished from ideational ones as they concern more universal, hard-wired ‘quirks’ of our mind that shape the way in which we think. Parsons (2007) suggests that psychological arguments imply irrationality because human behaviour is as much down to hard-wired instincts and affectations than to logical weighing of preferences. As a rule of thumb, we accept the proposition that innate psychological explanations for behaviour are more important than is often given credit, albeit less important than psychologists often think (Fiske 2002). As such, psychology might well provide important explanations for apparently irrational behaviour such as support for a park that is not considered beneficial. For example, Hsu *et al*. (2008) find that our sense of what is right is influenced by emotional reactions that are shaped by psychological responses such as sympathy and empathy. Thus, to understand responses to the external environment, such as support for particular distributional outcomes, we need first to understand something of human psychological predispositions.

**Fig. 1:**
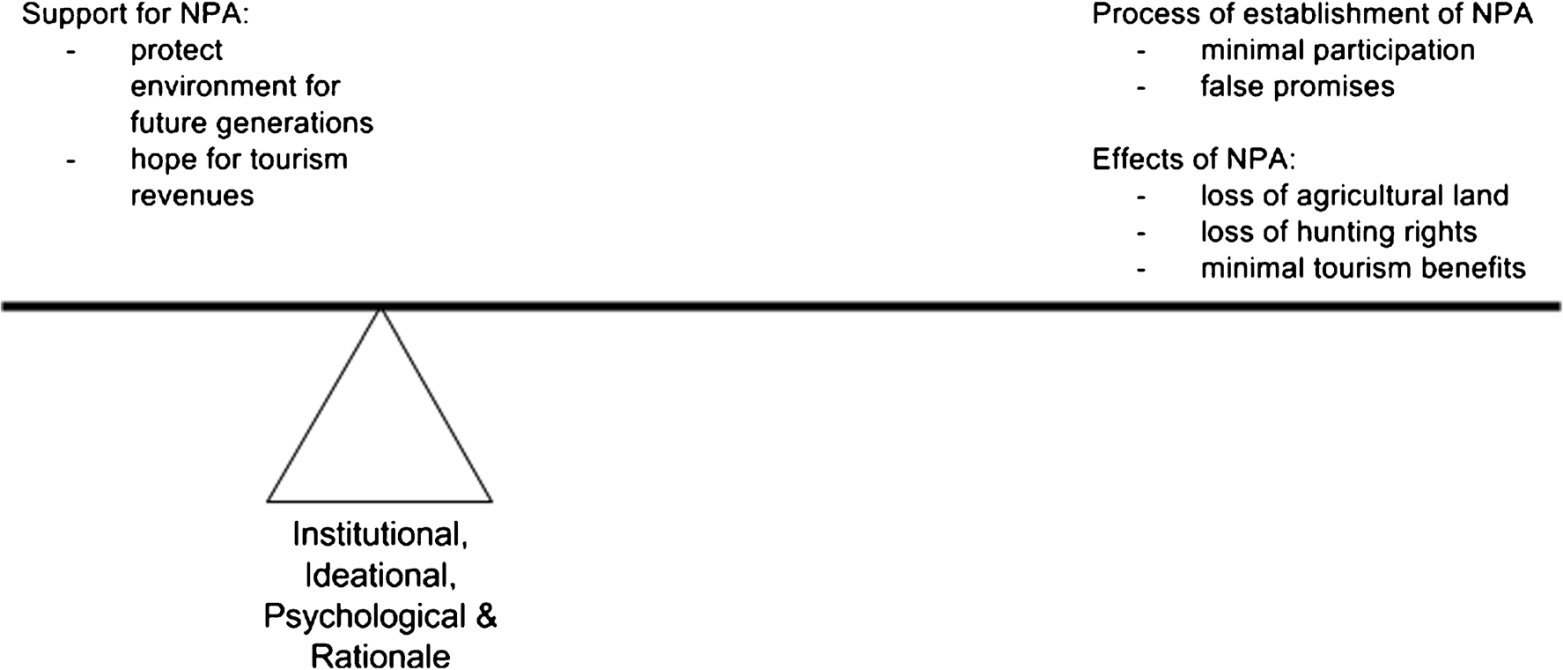
Villagers holding benefits and burdens of the NPA in balance through institutional, ideational and psychological means

We do not see these three different types of explanation as clear-cut or as necessarily competing with each other. Rather we see the potential for all to offer partial and perhaps complementary insights into a complex reality. We understand them as held in balance with one another, as shown in Fig. 1. For example, community conservation narratives have tended towards rationalist-institutionalist explanations, whereby villagers are predicted to respond to particular external institutional incentives by switching over from opposing to supporting the aims of parks (Jamal *et al*. 2003). As we have seen, however, the explanatory power of this type of argument is rarely sufficient and ideational explanations can help to explain particular place-based responses. For example, Robbins (1998) observed how resistance to a park in Rajasthan can only partly be explained by rational responses to imposed incentives and that it is also necessary to understand the local socio-environmental history that has shaped the way in which local people think about these institutions. In this case, the park authority-related institutions (ie the State) did not fit with customary institutions and were therefore perceived to be unjust and illegitimate, and were resisted. Satyal (2011) shows how local history shapes ideas about forest justice in Nepal, with notions of justice changing over time, from a religious-based tradition of *dharma,* shifting to emphasis on civil liberties in the mid twentieth century and then turning to the Maoist emphasis on equality after 1990.

Whilst ideational arguments can help us to understand local receptivity to institutions – how institutions become perceived and acted out in particular socio-ecological places - there may still be a missing element of explanation. In Laos, for example, we can understand deference to park rules in terms of disincentives to rule-breaking and in terms of historical events that have fostered high levels of dependence on state authority. Although such inter-linked institutional and ideational explanations are clearly important, we are not satisfied that they adequately explain apparently inconsistent beliefs, where the park is viewed as negatively impacting on lives but is also viewed positively. Psychological explanations might contribute to our understanding here, especially as the way humans resolve contradictory beliefs or values has been a major area of inquiry since Festinger’s (1957) work on cognitive dissonance. Amongst other things, this work seeks to understand how people rationalise their preference for autonomy in situations where rule compliance is effectively forced upon them.

### The Link to Legitimacy and Justice

Recent progress in understanding support for environmental management interventions has paid considerable attention to the notion of legitimacy and to associated ideas of fairness and justice (Pascual *et al*. 2014). We do not see the current analysis as departing from this focus, but rather as enriching it. van der Toorn *et al*. (2011, 127–128) theorised that the legitimacy of authorities is derived from “(a) the fairness of the actions by which they exercise their authority, (b) the favorability of the outcomes that they dispense, and (c) the degree of outcome control that they are seen as possessing or, conversely, the degree to which the perceiver is dependent upon them”. Others have identified connections between these different determinants of legitimacy, such that legitimate procedures are more likely (or even assumed) to lead to legitimate outcomes (Marion Suiseeya and Caplow 2013; Myers *et al*. 2016).

Environmental and social justice scholars identify similar elements that, individually or in combination, constitute claims about justice and injustice, and thus claims to legitimacy. Whilst *distributional* concerns have traditionally dominated struggles over environmental injustice (Schlosberg 2007; Walker 2012), there has been increasing emphasis on the importance of *procedural* and participatory concerns (see Sen 1999; Schlosberg 2007; Fraser 2009) and to the *recognition* of different cultures, values and rights (Taylor 1994; Fraser and Honneth 2003; Myers and Muhajir 2015). As with van der Toorn’s dimensions of legitimacy, these different elements are seen as intertwined, in the sense that failure to recognise cultural difference is likely to be linked to weak participation; and unjust decision-making procedures are unlikely to lead to just distributional outcomes (Walker 2012; Martin *et al*. 2013).

Explaining perceptions of legitimacy and justice would appear the natural academic territory for institutional and ideational arguments. But when we review again the kind of puzzle we are seeking to explain,we can see that psychological explanations can potentially enrich our understanding. In our Laos study site, expressions of legitimacy and fairness appear (superficially at least) to predominate in the absence of many of the conditions for legitimacy or justice that we have just reviewed. To turn van der Toorn’s findings upside down, we encounter real world situations in which authority is viewed as legitimate even where it is (a) exclusionary, (b) dispenses unfavourable outcomes, and (c) allows little or no autonomy to control those outcomes.

Looking to theories of psychology, this might be seen as an example of cognitive dissonance, where an individual holds two or more contradictory beliefs, or has to behave in a way that contradicts a belief or preference (Festinger 1957). Where such cognitive dissonance exists, it is proposed that we are psychologically predisposed to find a way to restore consistency. For example, if we are forced to comply with something (park rules) that we believe to lead to bad outcomes for us, we hold inconsistent beliefs and suffer dissonance. To overcome that dissonance, one common strategy is to re-evaluate our belief (Festinger and Carlsmith, 1959), i.e. to reconsider whether the outcomes of the park are really so bad. Jost *et al.* (2010) describe the phenomenon of ‘system justification’, whereby people are highly motivated to legitimise social institutions even where these ostensibly affect them adversely. The authors argue that:

“there are profound psychological factors that motivate individuals to accept, even support, the existing social system, even if that system entails substantial costs and relatively few benefits for them individually and for the community as a whole” (Jost *et al*. 2010, 173).

A primary motivation to justify adverse circumstances and systems is the potential risk and unpredictability that resistance might bring (Jost and Hunyady 2005). Sen’s (1999) work on adaptive preferences and autonomous wants is also relevant here. He shows that individuals adapt their preferences in response to social, economic and political factors that limit their choices. To hold on to preferences that can never be satisfied is not conducive to wellbeing and so we are psychologically predisposed to adapt expectations downwards in the face of diminished autonomy – a form of system justification, on which we will shed light later in this paper.

As we have said, we prefer not to assume primacy for any one type of explanation, and we don’t expect psychology alone to explain the circumstances in which people adapt their beliefs about parks to fit with exogenous circumstances. Indeed, part of the story we tell from Laos is not about people resolving opposing motivations through system justification and adaptation of preferences, but of a more complex situation in which people continue to hold apparently opposing views, due to the multiple ways in which conservation effects them. Institutional, ideational and psychological experiences are productive of one another and therefore must be considered together in order to better understand the paradox. Our interest is in understanding multiple costs and benefits and how these are legitimated, justified and opposed. We are particularly interested in how the same respondents could claim to support the park, despite the multiple hardships also claimed. Broadly speaking, we find evidence for system justification and therefore a role for psychological explanation. However, we also see this as connected to responses to institutional constraints and in particular the deference to state authority that is at least partly explained by high levels of dependence on the state. High levels of deference to the state are also partly understood in ideational terms, in relation to particular local histories of conflict and the post-conflict state.

## Methods

We consider institutional, ideational and psychological elements of explanation through an empirical focus on how local people think and act in response to selected procedural and distributional aspects of the Nam Et-Phou Louey NPA. The procedural aspect focuses on decision-making processes surrounding the gazetting of the NPA. Distributional issues focus on access to land, rules about hunting, collection of non-timber forest products and the provision of livelihood support.

**Fig. 2:**
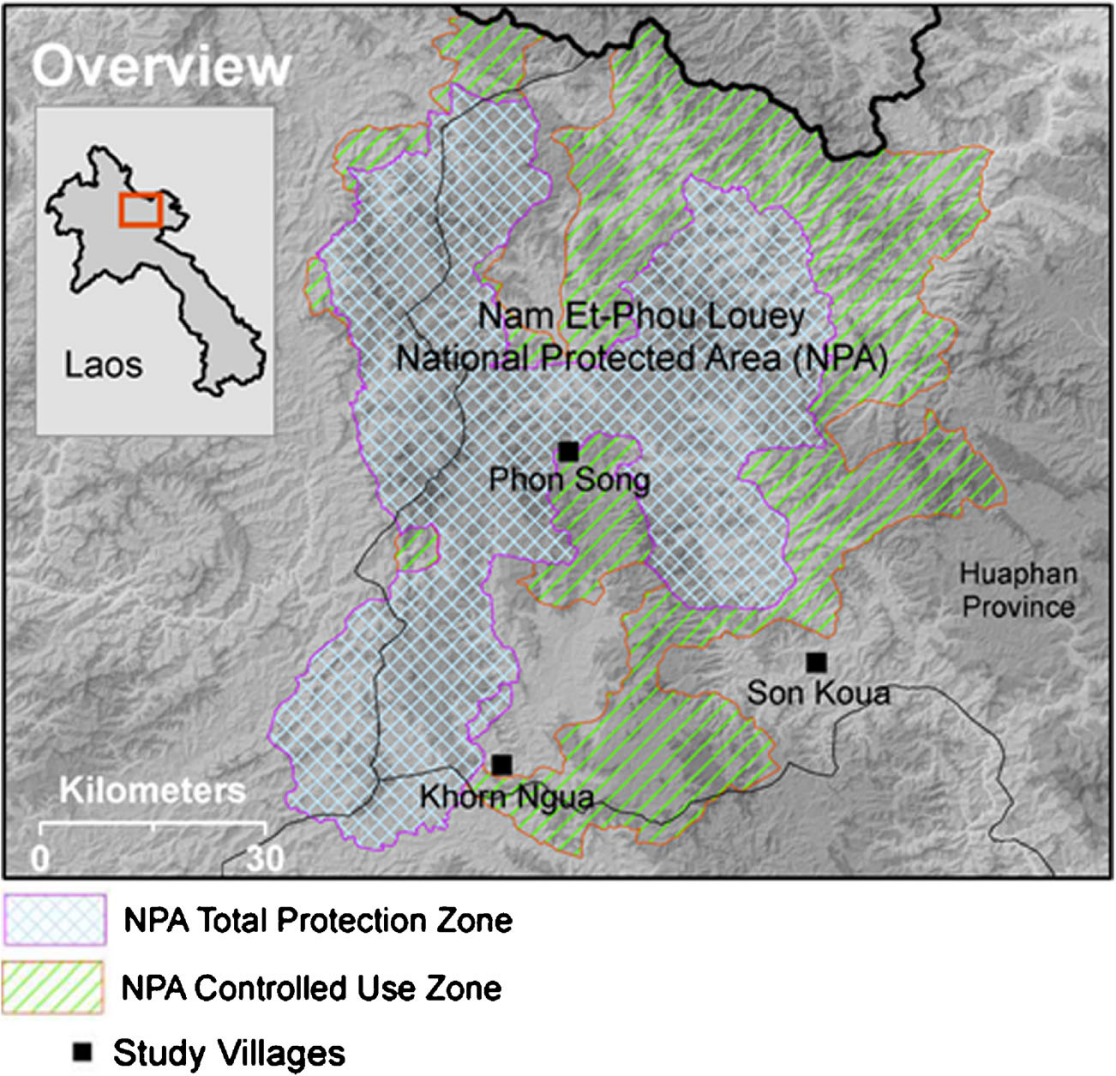
Map of Nam Et-Phou Louey NPA and case villages. Source: Project personnel Kenneth Grogan

There are 91,500 people in 13,600 households and 283 villages living within and immediately surrounding Nam Et-Phou Louey NPA (Nam Et-Phou Louey 2015). The NPA was established in 1993 with an area of 422,900 ha (Johnson 2013).^1^ Seventeen other NPAs were created in that same year by Prime Minister’s decree 164 (Robichaud *et al*. 2001). In 2008, the NPA was expanded (Bourgoin *et al*. 2013) to 595,000 ha (Johnson 2013) and is recognised as having high conservation value, being home to numerous endangered species (Johnson *et al*. 2016) (Fig. 2).

Relocation was fundamental for the establishment of the NPA, as it has been in the creation of other NPAs in Lao PDR (Evrard and Goudineau 2004). Evictions and relocations have been carried out as recently as 2007 and have lacked consultation with communities (Watts *et al*. 2011).

We selected three villages bordering the NPA that have similar histories of relocation, but have been impacted by the NPA to varying extents. Phon Song village is largely bordered by a ‘total protection zone’, Khon Ngua, village borders a ‘controlled use zone’ with greater resource access, Son Khua village also neighbours a ‘controlled use zone’ but also has an ecotourism scheme which employs villagers and distributes benefits to them. We expected that these different access regimes would lead to different experiences with the NPA.

We conducted semi-structured interviews with 100 households in Khon Ngua (30), Phon Song (30) and Son Khua (40) villages asking a range of questions pertaining to livelihood activities, land use change, wellbeing, and the NPA. We selected households at random from lists maintained by village heads. Either adult male or female were asked to take part, based on convenience, and sometimes both participated. We followed up household interviews with 30 more detailed life history interviews, to gain more in-depth data from a smaller sample, which was also randomly selected form the completed household interviews. Interviewers spent several weeks in the villages and engaged in discussions with a broad range of villagers prior to commencing formal interviews. This allowed a familiarity between respondents and interviewers, and elucidated the position of the interviewers as not aligned with the State or park authorities.

Our findings place considerable emphasis on what respondents tell us about their feelings towards the park and its rules. This raises a concern about how consistent these responses are with what respondents really think, because there is a risk that responses could be tailored to what respondents think they want the interviewer to hear; avoid confrontation with NPA authorities; and specifically in Southeast Asia, to employ deception as a form of resistance (Scott 1987). Whilst we cannot completely eliminate such phenomena, we invested considerable time into building a rapport and familiarity with people in the communities, link our survey data to in-depth interviews, and triangulate questions within data collection instruments.

After we had the interview transcripts translated, we coded the responses in QSR NVIVO 10 for Mac using data-driven nodes, with specific focus on the formation and management of the NPA, perceptions of ‘fairness’ of outcomes of the NPA, land use change, environmental services and disservices, and livelihoods. We analysed what respondents expressed about the NPA, what their thoughts were about the impacts of the NPA on their lives and how they rationalised these claims. We analysed the coded transcripts to gain insight into notions of justice and perceptions of legitimacy among respondents, differentiated by wealth categories derived using a Multidimensional Poverty Index (MPI)^2^^,.^ 3 Disaggregation by gender and age was not possible because the household data were sometimes supplied by multiple members of the household together.

## Results

### Institutional Explanations

Across the three villages, 100% of respondents stated that they support the NPA. This is not to say that everyone agreed with all the rules and regulations around the NPA, but that they agreed it was important to protect the forest and accepted that the NPA is a legitimate way of fulfilling that objective. Respondents state that establishment of the NPA is good because it provides an opportunity for animal populations to increase and deforestation has been stemmed. As we show in the following sections, respondents also had myriad complaints about the NPA. From an institutional perspective we might expect their support for the park to be explained by self-interested responses to a range of incentives shaped by the rules and procedures of the park. We begin by considering that this self-interest might be linked to economic incentives arising from the NPA and then consider that participation might itself be a form of incentive; i.e. that people value local inclusion and local control in natural resource management.

### Economic Incentives

There are three main economic concerns discussed in the interviews: access to agricultural land, hunting and non-agricultural employment. Land remains the most important asset for livelihoods in all three villages, producing rice for subsistence and maize for sale to the Vietnamese livestock industry. The surge in commercial maize production has driven rapid transitions in livelihoods that include rising average incomes. Virtually all respondents are pleased with this trajectory and it helps to underpin perceived legitimacy of government planning. However, the NPA, in combination with population growth, has led to increased land scarcity and some now report the need to switch back from maize to rice in order to restore food security. Interestingly, it is the non-poor category of households who are most likely to state that land is insufficient (81%) and the chronically poor group who are least likely to report this problem (50%). This is because access to land for the poorest is more constrained by lack of affordability than by lack of physical availability. As one respondent explained, “the people who have more land are the people who have money, they just buy the land for keeping, but we are a new couple, so we don’t have money.”

Nevertheless, lack of land availability has led to hardship for the poor, most notably in Phon Song village, which is next to the total protection zone and has faced the greatest restriction. Here, both average land holding (1.15 ha) and average annual household farming incomes (5.63 million Kip) are statistically lower than the other villages, whilst household debts are significantly higher. Furthermore, the loss of land for rice farming has led to a large increase in food insecurity with a rise from 15% (2004) to 43% (2014) of Phon Song households declaring insufficient self-production to meet household rice needs and increasing difficulty affording to buy in the shortfall. As one Phon Song respondent from a poor household explained, “after the NPA came, the land is too small… It is good to have the NPA, but the paddy rice isn’t enough for people.”

Avoiding hunger remains a prominent concern for many villagers. The NPA restrictions on hunting are widely cited as contributing to the difficulty of meeting food requirements:

“[in the past] we could hunt wild animals and find much more fish in the river, so we ate more in the past. But now the NPA came we have much less meat and there are less fish in the river too; the same with birds, wild chicken, rat, red cheeked squirrel. It was always hard to find bigger animals though. There was not enough to sell any, now or in the past.”

Even though some respondents suggest that they have less food to eat than before the NPA was created and meat eating is restricted to “only rats”, there is still widespread support for limits on hunting. Overall, 91% respondents claim that there should be some sort of restriction on hunting, as either a total ban (48%), just in the NPA (37%) or of specific species only (6%). Differences between villages point to rational economic determinants of support for NPA rules. In Phon Song, where the NPA has impacted most strongly on resource access, and where food shortage has become most common, support for a total ban on hunting is the lowest at 24%. In Son Khua, the village where there is investment in an ecotourism enterprise and livelihoods are the most diversified, support for a total hunting ban is the highest at 53%. The poorest households tend to be most supportive of the ban on hunting in the NPA. This might seem counter-intuitive (from a rational economic perspective) but we think this is explained by their limited time and resources to hunt, the unaffordable level of fines, and the importance the poor place on consistent application of rules (the worst situation for the poor is where they have to comply with restrictions whilst more powerful households find ways around them, with many poorer households citing differences in household ability to pay fines).

There is some evidence that support for the NPA was encouraged through the promise of employment and investment in local services and infrastructure by the State. But these potential benefits have mainly failed to materialise. In Son Khua, the expectation was highest due to the ecotourism business. However, some of these jobs (such as transportation) went to outsiders and the village-based jobs are few because of the small number of tourists. Thus, whilst the promise of future economic benefits might have initially been a cause to support NPA formation, this incentive appears to have waned.

### Participation as Incentive

Our data provides very little evidence that support for the NPA was driven by appreciation for participatory procedures and inclusive governance. We consider participation as a mechanism that can contribute to support for PAs, especially if the voices of local actors are incorporated into PA governance (Scherr *et al*. 2003; Larson and Petkova 2011), however, participation is no guarantee of support for the park; nor is it a prerequisite for the success of conservation from biophysical perspectives (Brockington *et al*. 2008). Respondents across the three villages consistently reported a lack of any real participation in the planning of the NPA, as summarised by a Phon Song respondent who said, “there wasn’t anything we could say, they had already decided how it would be.” In all three villages, officials came to the village, explained that there would be an NPA, where the borders were, and the activities that would no longer be permitted both within the NPA and outside its boundaries. As a respondent from Khon Ngua explained:

“when the NPA came they just set a meeting for the whole village and told us the objectives of why they came. They came to promote conservation. They said if we continue with the same livelihoods and hunting there will be no wildlife and we need to save it for future generations. They just set the rules, we didn’t negotiate with them. But we don’t have any problems with the NPA.”

Interviews yielded consistent evidence that the district government came to inform, rather than to engage in discussion about the formation of the NPA. Some also reported wariness about contradicting government agents, suggesting that “it might be bad for them if they chose to speak up.”

One exception to this was in Phon Song, where several respondents suggested that after protest, the NPA authorities moved the borders to accommodate specific parcels of agricultural land. Here, the original boundary markers were clearly untenable as they passed too close to the edge of the village settlement and left swathes of farmland in the total protection zone. In Son Khua and Khon Ngua, villagers also found themselves surprised and often unhappy with the location of boundaries. A Son Khua respondent explained that prior to boundary markers appearing “the people didn’t have any problem”, but that “only when we know the border, we had to move to do upland rice [outside the NPA].” Indeed, half of all respondents brought up the issue of the NPA boundaries. Some Khon Ngua respondents reported that authorities invited villagers to contest borders if they had land under paddy cultivation within the NPA. At the time of interviews, however, these requests for changes had received no response. Similarly, ten respondents from Son Khua said they had contested the boundary but had failed to elicit any response from the NPA authority. In Phon Song, despite the initial change to the boundary in 2008, there had still been further calls for changes, for example from a family who told us that their land had been incorporated into the NPA during this change. These further requests for change had gone unanswered.

Despite the lack of responsiveness to local claims, support for the NPA is systematically expressed in procedural rather than distributional terms, with emphasis on the importance of supporting the government and the associated rules. A respondent from Son Khua explained that “I am not sad [about the restrictions of the NPA] because it is a plan of the government. We are people, so we understand the plan of government”. Another from Khon Ngua gave the simple explanation of why villagers agreed with the plan for the NPA: “because they had the announcement letter with them.”

Throughout our interviews, there was strong emphasis on the importance of obeying rules. For example, 90% of those who agreed it was right to fine people for hunting offered the reason that it was against the rules. This could in part be explained in rational, institutional terms. Punishment takes the form of jail sentences and fines, with the latter most common for minor offenses such as hunting small animals. Several respondents did speak about the difficulty of paying the fines as being a disincentive and said they would consider hunting in the NPA if they could afford the fine. However, the economic incentives linked to rules do not adequately explain the strong emphasis placed on obeying them. For example, by far the most common reason for supporting fining other people for hunting is simply that “there are rules”, with no reference to associated economic costs and benefits or to concerns about unfair gain through e.g. free-riding. When explaining their own behaviour, most respondents did not express rule adherence as an economic decision. As one respondent commented, “we don’t think to hunt wildlife again, because it is a sin”. The idea of ‘sin’ seems rooted in notions of injustice and illegitimacy. Another respondent qualified his agreement with the NPA rules as fair as follows: “when they came and set up all the rules, I thought it would be difficult to find enough to eat. They said no hunting [with guns or in the NPA], no chopping trees….We just had to follow the rules.” Is it fair? “Sure, it’s fair.” To more fully understand support for the NPA, and in particular, understanding how this is articulated as support for rules, we need to explore the importance of historically rooted relationships with the state, which we have categorised as ideational explanations.

### Ideational Explanations

In terms of their cultural values and identities participants were primarily farmers who had practised shifting rice cultivation in upland fields for many generations. This land use practice created a mosaic of forest habitats from which people foraged a range of products for food, construction, medicine, fuel and tools. Beliefs, rituals and festivals of both animist and Buddhist religions were tied to features in the landscape through the presence of spirits and to the climate-regulated farming cycles. As one respondent expressed:

“When we go to the forest it is part of our culture. We need to collect food for the household, but it is important to collect things for the village spirit. When we trap mice and things we sometimes give them to the spirit….. I believe that if you do that the spirit protects me from the bad things, it ensures a good harvest and means when we go to the forest we will be protected from bad things. It is the same as it has always been. All of the village come together to celebrate before farming. We do it in the first week of March every year, depending on the lunar calendar.”

Social practices and cultural values were also changing for a number of reasons, including: the aftermath of war and associated insecurity; the relocation of the rural population from within the forest to be situated alongside roads, which served to reduce inter-ethnic conflict and initiate development; and economic transition from a subsistence economy to a more diversified and marketised economy connected to businesses and consumers in China and Vietnam. Like many in the region, the three villages in this study had been newly formed during the 1980s and 1990s when government resettlement programs sought to bring people out of their original villages, often deep in the forest, to live next to newly developed road networks to enable easier communication, service provision and trade. The move to relocate away from remote areas of ongoing conflict, to gain greater access to facilities such as health centres and schools, and to potentially diversify livelihoods was viewed unanimously by participants as positive. Alongside agrarian change, investment had been made in infrastructure and each village now boasted a school, nearby health centre, access to electricity and a clean water supply.

The top-down decision-making and some of the negative outcomes of the protected area may be accepted by villagers because their own priorities have become better aligned with conservation goals. Participants described their priorities as being security, diversification of livelihoods and income generation while also maintaining forest ecosystem services for a range of current and future provisioning, regulating and associated cultural ecosystem services. The State exercised further control over farming activities in village land through land-use planning exercises, which explicitly aimed to eradicate shifting cultivation. Despite the limited participation and only cursory consultation, villagers commonly describe these processes as ‘fair’. This can be partly explained by the prioritisation of security by people who have experienced severe, long-term conflict in their recent histories. Villagers appeared content to defer decisions about the NPA to district or provincial authorities for the purposes of maintaining order and avoiding any conflict between different groups within or between villages. Several respondents referred to conflict before they were relocated and a desire to avoid such conflict again. This prioritisation of security makes other problems seem less pressing, and may make them less willing to contest other perceived economic injustices. As one respondent from Phon Song mentioned,

“The relations between villagers are not good at the moment, there are too many ethnic groups here so the district doesn’t want to come and deal with us very much here. It’s not like people from my old village are separate to those from other villages but there is conflict between some of the groups.”

Additionally the change to cash cropping incentivised the clearance of large areas of old fallow land. Villagers commonly conveyed a sense of guilt at their unsustainable use of soils for short term gain. As one respondent stated,

“Since the maize company came here we cut lots of trees, more than we would for planting rice. The rice planting is only to have enough to eat but for the maize you simply can’t get enough land for growing it.”

The NPA therefore served a purpose to limit this exploitation and preserve local climate, water purity and valued forest resources important to maintain elements of their culture. For these reasons the demarcation of NPA boundaries and increasing penalties for agricultural encroachment or for hunting certain animals were strongly supported by locals. Whilst access to culturally significant resources had been restricted, villagers were able to continue collecting these resources outside the protected area. Activities such as hunting, basket weaving and trap-making were often stated by villagers to be continued out of economic necessity, yet they also recognised the social and cultural value of maintaining those skills across generations. Rice baskets, sieves and many other items continued to be made and used without replacement by modern alternatives despite higher incomes, greater rule and time restrictions and reduced availability of forest resources. People from 24 of the 100 households reported that they still actively hunted in groups for large animals such as wild pigs and deer. Many more retain their weapons in order to continue hunting despite high potential fines for being caught in possession of a rifle. However in another sense the rapid changes in people’s values, identities and aspirations was also leading to increased dissatisfaction with the outcomes of conservation. In order for persuade villagers to accept the boundaries and rules of the NPA, they were promised support for farm productivity and marketing. These promised changes to livelihoods would enable villagers to align their actions with a discourse of ceasing shifting cultivation in favour of more market-oriented and permanent agriculture. Yet negligible support materialised. As one respondent framed it, “Simply sticking to the ideal that we can continue growing maize in an area is not realistic for us. New types of cash crops that fit the realities we are facing are essential to create a meaningful and sustainable solution.”

As villagers became dissatisfied with the lack of promised support, they began formal efforts to claim back land from the NPA, some of which had been the site of villagers’ previous villages or had been farmed in the past. These claims had lain dormant but were being rekindled due to the premium attached to flat, productive land that could support permanent farming. These lands were viewed by villagers as important to enable the switch away from shifting cultivation, in addition to the underlying cultural connections to those specific places. Relatively poor households with limited social resources felt further impeded in making progress towards their aspirations by the unequal access to land. While they could not risk breaking rules to cultivate land within the NPA, wealthier households could and often extended their land and income-earning potential without punishment. Some poorer households therefore voiced dissatisfaction with the inconsistency with which NPA rules were applied and the inaction of authorities in promoting a more equal distribution of cultivable land between households.

Hence dynamic ideational factors explain how the conservation strategy can be supported as legitimate and fair and how it can be perceived as illegitimate. On the one hand supporting the authority associated with the NPA resonates with villagers’ priority for peace and stability, whilst also promising alignment with their aspiration to develop more modern agricultural livelihoods. But on the other hand promises have not been realised, leading to disenchantment with the NPA and attempts to re-establish access to some gazetted lands. Thus, the approach reveals tensions between tendencies to accept state interventions, particularly based on associated promises of support for livelihood transitions, and subsequent disappointment with a lack of realisation of support and inability to meet contemporary aspirations. In the next section we explore some of these tensions through psychological explanations.

### Psychological Explanations

The fact that all 100 respondents expressed support for the NPA remains only partly explained. Fear of fines and imprisonment certainly provides a disincentive to break the rules of the NPA, and recent social, economic and ecological histories help to explain a predisposition towards more positive support for state rule-making, and in particular rules that align with concerns about security and with the prevalent development discourse of agricultural modernisation and livelihood diversification. However, our brief exploration of institutional and ideational explanation does little to explain away the simultaneous presence of support and resentment toward the NPA. We therefore try to shed more light on these apparently contradictory behaviours, this time from a psychological perspective, which deals more with how ideational and institutional experiences are internalised by respondents.

Our study did not set out to explore psychological determinants of views about the NPA and so we have only used the ideas of cognitive dissonance and system justification as an ex-post attempt to explain an apparent paradox. Following cognitive dissonance theory, we tentatively frame the problem for villagers like this: they are compelled to follow the rules of the State, but doing so results in severely limited ability to benefit from forest resources. We offer this as a possible factor contributing to the predispositions of the people we interviewed and present our framing as one that requires further exploration. Having to support institutions that are detrimental to one’s own interests results in a form of psychological tension. Cognitive dissonance theory holds that people are predisposed to resolve such internal tensions rather than allow permanent dissonance. More specifically, resolution is achieved through ‘system justification’, which will typically involve a disposition towards internalising the positive outcomes of supporting the prevailing governance institutions. In our case, we propose that system justification involves being highly receptive to messages about the positive benefits of the NPA, in a way that internalises the ‘decision’ to support it. Looking for evidence of such system justification, the thing that stands out in our data is a tendency to readily agree and even amplify the environmental benefits of the NPA. As with many protected areas, park authorities make a considerable effort to ‘market’ such environmental benefits to local people, providing what is sometimes called environmental education or community sensitisation. As elsewhere, the environmental benefits that are marketed to local people include climate and water regulation, wildlife protection and opportunities for related industries such as ecotourism.

When asked about the benefits of the NPA our respondents mentioned this range of potential outcomes, including better climate and more oxygen (37 respondents, 48% from Son Khua) and improved water supply (22 respondents, 59% from Son Khua). However, the two potential benefits that were particularly prominent were protection of tigers and nature tourism. Regarding tigers, the NPA authorities had undertaken a specific marketing campaign using the slogan that “we are proud to have tigers” (as witnessed on posters around the park). The ‘we’ in this slogan referred to the community and thus the NPA authority sought to represent the views of the community. We suspected this was a mis-representation of local opinion but when asked about this statement, all but one respondent agreed with it.

Even those who had never seen a tiger support their protection. One respondent said, “I think I would like to see [a tiger], because I have never seen one before. I have only seen them in pictures”. This pride in tigers and support for their protection is relatively new. For example, in Phon Song villagers told us stories from the past, including an occasion on which a tiger came right into the village and ate from a cooking pan. When asked how they responded, they said they tied a pig to a tree, hoping the tiger would return so they could shoot it. Indeed respondents made frequent mention of tigers killing livestock and several respondents described fears of being attacked by a tiger. But nowadays the economic and personal threats posed by tigers do not lead to the simple response that tigers should be killed. As one respondent said, “I am afraid the tiger will bite me, [but] I am happy [that we are conserving them].”

This change in response is not only a result of marketed ‘pride’ in tigers but in the marketing of associated economic benefits that can flow from tourism. Villagers have been primed to pin their hopes on tigers, and conservation more generally, to bring tourism revenues, something that they were told by NPA authorities would come after the establishment of the NPA. “I think if there are a lot of tigers, there will be more tourists come to visit, because we have conservation of wildlife”. More than half of the respondents in Son Khua mentioned either present or future benefits from tourism when posed an open question about the benefits of the NPA. Substantially fewer mentioned tourism in Khon Ngua and Phon Song where tourism has not yet been developed or actively marketed by the NPA authorities. Nonetheless, tourism was already being thought about in other villages, for example one respondent in Khon Ngua expressed his optimism in saying, “if we can conserve [the forest], it will be famous. There will be a lot of tourists who come to visit.”

As the most advanced village in terms of tourism development, Son Khua had not only experienced strong advocacy regarding the benefits of tourism but had also begun to experience some of the realities. It was already observed that tourism was insufficient as a basis for transforming livelihoods and only benefited a minority of people. However, the responses we received suggest that this reality check has done little to dampen continued receptiveness to the message that tourism benefits are a strong reason to support the NPA. Although respondents are so far not satisfied with the levels of tourism produced by the NPA, the allure remains even in the villages where NPA promises have yet to be fulfilled. Villagers demonstrate considerable optimism even when they have been given little reason to trust the State.

The question of most interest to us here is why are villagers so receptive to the positive environmental and tourism benefits marketed by the NPA authorities? One explanation for this would simply be to propose that the social marketing strategy of the NPA (see Saypanya *et al*. 2013 )has been highly effective. In other words, we could employ an explanation based on factors largely external to the community – the quality of the message delivered to them. But this would be to underappreciate the capacity of local people to critically process marketing and overlook the psychological factors that may enhance receptivity to advertising. In particular, that marketing can be internalised as a means of resolving cognitive dissonance, something that Thøgersen (2004) shows in his work on environmentally responsible behaviours among consumers.

We propose that the perception of positive outcomes among villagers is partially a testament to the efficacy of the marketing conducted by the NPA authorities, but that villagers were also susceptible to accept such messaging from a psychological perspective, explained by system justification as a means to resolve cognitive dissonance (after Jost *et al*. 2010). The losses to livelihoods that we have already explained and the exclusion of villagers from decision-making around the NPA, combined with historical territorial wars among ethnic groups followed by relocations by the State, render them eager to view the NPA in a positive light. The ‘we are proud to have tigers’ marketing campaign shows that the NPA is willing to represent the villagers’ interests without consultation and market to them how they ought to feel about conservation. As our analysis shows, people have many reasons to not be happy that there are tigers in the area. They articulate reasons that they fear and resent tigers.

## Discussion and Conclusions

The starting point for this paper was the observed paradox that people living near to parks are often supportive of their purpose even when this appears to go against some of their key livelihood interests. Some previous literatures have sought to understand support for parks in terms of the different types of benefits that people attain, including both economic and governance preferences (e.g., Khatun *et al.* 2015) whilst others have related support to the extent to which people perceive the park to be fair or legitimate in relation to procedures and distributional outcomes (eg. Pascual *et al*. 2014). Here, faced with the paradox of claimed legitimacy, despite the apparent lack of procedural or distributional fairness, we have explored the complex, multiple and sometimes contradictory views and behaviours in response to the park. Whilst this has been an exploratory study we think it provides a promising direction for seeking a fuller understanding of how notions of fairness and legitimacy are constructed.

Institutional explanations show that the effects of the park (the incentives and constraints) are themselves multiple, for example hunting restrictions may be offset by income from ecotourism. These multiple institutional ‘signals’ help to explain the multiple and complex views surrounding support for the park. Furthermore, local responses to economic rules are patterned by a high level of dependence on authority. The authoritarian institutions of state help to understand how rule adherence surfaces as a dominant theme. The authorities are feared and rules are followed not because they have popular support, but simply because they are the rules and are backed up by hierarchical power.

However, respect for rules and authority cannot be explained in institutional terms alone and requires understanding of local historical context. Ideational explanations seek to understand how responses to institutions are mediated by critical contextual factors such as war and insecurity, state-sponsored modernisation programmes and resettlement. For example, the recent history of conflict and migration helps to explain the prioritisation of security and stability, even where this requires support for institutions that runs counter to everyday economic interests. Hence there are place-bound contexts that help to explain the way in which communities interpret and think about imposed institutions. Even though we observed forms of resistance to park rules, deference still figures highly. Respondents articulate many problems arising from both procedures and outcomes of the NPA, providing plenty of motivation to resist the NPA. These negative views co-exist in the minds of villagers alongside the overriding compulsion to obey and support the rules of the park. But because of this tension, the apparent ‘support’ for the NPA can be seen to be weak and, as recent resurgence of claims to land in the NPA attests, is sensitive to changes in values or aspiration.

Adding further to this complex picture, we suggest that psychological insight based on system justification might contribute to understanding our opening paradox. Whilst an ideational perspective helps to understand rule adherence, it does not explain why we see regular patterns of justification for the park, based on perceived environmental benefits such as regulatory ecosystem services (hydrological and climate), love for wildlife (especially tigers), tourism, and the ecological aspects of the environment peculiarly explained in technical terms like ‘oxygen’. To understand these reasons, we have to look to the success of social marketing of the NPA. And to understand this success, we propose that hard-wired psychological responses - notably the human need to resolve cognitive dissonance - could be used to explain local receptivity to this marketing. Tentatively, we hypothesise that internalising these marketing messages provides the opportunity to justify the system that people are irrespectively compelled to support. We argue that much of the socio-political work on conservation, including our own, overlooks psychological theories and perspectives of analysis.

Social scientists will not be surprised by yet another call for the appreciation of complexity of interactions among the State, local communities, and forests, but we emphasise that the use of institutional, ideational and psychological devices can provide a meaningful toolkit to parse these complex interactions into bundles conducive to analysis and provide explanatory power to sometimes labyrinthine paradoxes. What seemed to us at first as contradictory data – that villagers both thought the park was fair and unfair at the same time – can be explained using these devices to make sense of these contradictions.

## Footnotes

1 Approximations of the actual size of the NPA vary widely (Robichaud *et al*. 2001).

2 See http :// hdr . undp . org / en / content / multidimensional - poverty - index - mpi. We also recognise the weakness of using a global tool to measure poverty, but find it useful here to understand roughly how levels of poverty affect perspectives on the NPA.

3 Not poor (MPI 0–0.19); vulnerable to poverty (MPI 0.20–0.33), poor (MPI 0.34–0.50) and chronically poor (MPI 0.51–1.00). For simplicity we, sometimes use least poor households (MPI 0–0.33) and poorest households (MPI 0.34–1.00).

## References

[CR1] Allendorf, T. (2007). Residents’ Attitudes toward Three Protected Areas in Southwestern Nepal. Biodiversity and Conservation 16(7): 2087–2102. Springer.

[CR2] Allendorf, Teri, Swe, K. K., Oo, T., Htut, Y. E., Aung, M., Allendorf, K., Hayek, L.-A., Leimgruber, P., and Chris Wemmer. (2006). Community Attitudes toward Three Protected Areas in Upper Myanmar (Burma). Environmental Conservation 33(04): 344–52. Cambridge University Press.

[CR3] Baker, J., Milner-Gulland, E. J., and Leader-Williams, N. (2012). Park Gazettement and Integrated Conservation and Development as Factors in Community Conflict at Bwindi Impenetrable Forest, Uganda. Conservation Biology: The Journal of the Society for Conservation Biology 26 (1): 160–70. Wiley Online Library. 10.1111/j.1523-1739.2011.01777.x 22044616

[CR4] Béland D (2009). Ideas, Institutions, and Policy Change. Journal of European Public Policy.

[CR5] Blomley T (2010). Development and Gorillas? Assessing Fifteen Years of Integrated Conservation and Development in South-Western Uganda.

[CR6] Bourgoin J, Castella JC, Hett C, Lestrelin G (2013). Engaging Local Communities in Low Emissions Land-Use Planning: A Case Study from Laos. Ecology.

[CR7] Brockington D, Duffy R, Igoe J (2008). Nature Unbound: Conservation, Capitalism and the Future of Protected Areas.

[CR8] Bush, G., and Mwesigwa, R. (2008). Costs and Benefits from Protected Areas. Action Research Project--Bwindi Impenetrable Forest National Park, Uganda, Unpublished Report, CARE International in Uganda.

[CR9] Carstensen MB, Schmidt VA (2016). Power Through, over and in Ideas: Conceptualizing Ideational Power in Discursive Institutionalism. Journal of European Public Policy.

[CR10] Chhatre A, Agrawal A (2009). Trade-Offs and Synergies between Carbon Storage and Livelihood Benefits from Forest Commons. Proceedings of the National Academy of Sciences of the United States of America.

[CR11] Cinner, J. E., Huchery C., Aaron MacNeil M., Graham N. A. J., McClanahan T. R., Maina J., Maire E., *et al*. 2016. Bright Spots among the World’s Coral Reefs. Nature. https://www.ncbi.nlm.nih.gov/pubmed/27309809. 10.1038/nature18607 27309809

[CR12] Coleman, E. A., and Fleischman, F. D. (2012). Comparing Forest Decentralization and Local Institutional Change in Bolivia, Kenya, Mexico, and Uganda. World Development 40(4): 836–49. Elsevier.

[CR13] Daigneault P-M, Béland D (2015). Taking Explanation Seriously in Political Science. Political Studies Review.

[CR14] Evrard, O., and Goudineau, Y. (2004). Planned Resettlement, Unexpected Migrations and Cultural Trauma in Laos. Development and Change 35(5): 937–62. Blackwell Publishing Ltd/Inc.

[CR15] Ferraro, P. J. (2001). Global Habitat Protection: Limitations of Development Interventions and a Role for Conservation Performance Payments. Conservation Biology: The Journal of the Society for Conservation Biology 15 (4): 990–1000. Blackwell Science Inc.

[CR16] Festinger, L. (1957). A Theory of Cognitive Dissonance, Vol. 2, Stanford University Press.

[CR17] Festinger, L., and Carlsmith, J. M. (1959). Cognitive Consequences of Forced Compliance. Journal of Abnormal Psychology 58(2):203–10. psycnet.apa.org. 10.1037/h0041593 13640824

[CR18] Fiske, S. T. (2002). What We Know Now about Bias and Intergroup Conflict, the Problem of the Century. Current Directions in Psychological Science 11 (4). cdp.sagepub.com: 123–28.

[CR19] Fraser N (2009). Scales of Justice: Reimagining Political Space in a Globalizing World.

[CR20] Fraser, N., and Honneth, A. (2003). Introduction: Redistribution or Recognition? In Fraser, N., and Honneth, A. (eds.), Redistribution or Recognition? A Political-Philosophical Exchange, Verso, London.

[CR21] He J, Sikor T (2015). Notions of Justice in Payments for Ecosystem Services: Insights from China’s Sloping Land Conversion Program in Yunnan Province. Land Use Policy.

[CR22] High H (2014). Fields of Desire: Poverty and Policy in Laos, Challenges of Agrarian Transition in Southeast Asia.

[CR23] Hsu M, Anen C, Quartz SR (2008). The Right and the Good: Distributive Justice and Neural Encoding of Equity and Efficiency. Science.

[CR24] Hutton, J., Adams, W. M., and Murombedzi, J. C. (2005). Back to the Barriers? Changing Narratives in Biodiversity Conservation. Forum for Development Studies 32(2): 341–70. Taylor & Francis.

[CR25] Jamal T, Everett J, Dann GMS (2003). Ecological Rationalization and Performative Resistance in Natural Area Destinations. Tourist Studies.

[CR26] Johnson A, Sunderland TCH, Sayer J, Minh-Ha H (2013). Nam Et-Phou Louey National Protected Area. Evidence-Based Conservation: Lessons from the Lower Mekong.

[CR27] Johnson, A., Goodrich, J., Hansel, T., Rasphone, A., Saypanya, S., Vongkhamheng, C., Venevongphet, and Strindberg, S. (2016). To Protect or Neglect? Design, Monitoring, and Evaluation of a Law Enforcement Strategy to Recover Small Populations of Wild Tigers and Their Prey. BIOC 202 (C): 99–109. Elsevier Ltd.

[CR28] Jost JT, Hunyady O (2005). Antecedents and Consequences of System-Justifying Ideologies. Current Directions in Psychological Science.

[CR29] Jost JT, Liviatan I, van der Toorn J, Ledgerwood A, Mandisodza A, Nosek BA, Bobocel DR, Kay AC, Zanna MP, Olson JM (2010). System Justification: How Do We Know It’s Motivated?. The Psychology of Justice and Legitimacy: The Ontario Symposium.

[CR30] Karanth, K. K., and Nepal, S. K. (2012). Local Residents Perception of Benefits and Losses from Protected Areas in India and Nepal. Environmental Management 49(2): 372–86. Springer. 10.1007/s00267-011-9778-1 22080427

[CR31] Khatun, K., Gross-Camp, N., Corbera, E., Martin, A., Ball, S., and Massao, G. (2015). When Participatory Forest Management Makes Money: Insights from Tanzania on Governance, Benefit Sharing, and Implications for REDD+. Environment and Planning A 47(10): 2097–2112

[CR32] Larson AM, Petkova E (2011). An Introduction to Forest Governance, People and REDD+ in Latin America: Obstacles and Opportunities. Forests, Trees and Livelihoods.

[CR33] Lejano, R. P. (2006). Theorizing Peace Parks: Two Models of Collective Action. Journal of Peace Research 43(5): 563–81.

[CR34] Nam Et-Phou Louey. 2015. People of the Park - Nam Et-Phou Louey. *Nam Et-Phou Louey*. http://www.namet.org/about/people-of-the-park/.

[CR35] Marion Suiseeya KR, Caplow S (2013). In Pursuit of Procedural Justice: Lessons from an Analysis of 56 Forest Carbon Project Designs. Global Environmental Change: Human and Policy Dimensions.

[CR36] Martin A, Akol A, Phillips J, Sikor T (2013). Just Conservation? On the Fairness of Sharing Benefits. The Justices and Injustices of Ecosystem Services.

[CR37] Martin, A., Akol, A., and Gross-Camp, N. (2015). Towards an Explicit Justice Framing of the Social Impacts of Conservation. Conservation and Society 13(2): 166. Medknow Publications and Media Pvt. Ltd.

[CR38] Myers R., and Muhajir M. (2015). Searching for Justice: Rights vs ‘benefits’ in Bukit Baka Bukit Raya National Park, Indonesia. Conservation & Society 33(4).

[CR39] Myers, R., Sanders A., Larson A. M., Ravikumar A., and Rut Dini Prasti H. 2016. Analyzing Multilevel Governance in Indonesia: Lessons for REDD+ from the Study of Land Use Change in Central and West Kalimantan. 202. Occasional Paper. Bogor: CIFOR.

[CR40] Newmark, W. D., Leonard, N. L., Sariko, H. I., and Gamassa, D.-G. M. (1993). Conservation Attitudes of Local People Living Adjacent to Five Protected Areas in Tanzania. Biological Conservation 63(2): 177–83. Elsevier.

[CR41] North DC (1990). Institutions, Institutional Change and Economic Performance.

[CR42] Oates, J. F. (1995). The Dangers of Conservation by Rural Development--a Case-Study from the Forests of Nigeria. Oryx: The Journal of the Fauna Preservation Society 29(02): 115–22. Cambridge Univ Press.

[CR43] Oldekop, J. A., G. Holmes, and W. E. Harris. 2015. A Global Assessment of the Social and Conservation Outcomes of Protected Areas. Conservation. Wiley Online Library. http://onlinelibrary.wiley.com/doi/10.1111/cobi.12568/full. 10.1111/cobi.12568 26096222

[CR44] Orloff AS, Palier B (2009). The Power of Gender Perspectives: Feminist Influence on Policy Paradigms, Social Science, and Social Politics. Social Politics: International Studies in Gender, State & Society.

[CR45] Ostrom, E., and Nagendra, H. (2006). Insights on Linking Forests, Trees, and People from the Air, on the Ground, and in the Laboratory. Proceedings of the National Academy of Sciences 103(51): 19224–31. National Acad Sciences. 10.1073/pnas.0607962103 PMC1838564 17088538

[CR46] Parsons, R. (2007). The Emergence of Institutionalised Social Dialogue in South Africa. The South African Journal of Economics. Suid-Afrikaanse Tydskrif Vir Ekonomie 75(1): 1–21. Blackwell Publishing Inc.

[CR47] Pascual, U., Phelps J., Garmendia E, Brown K, Corbera E, Martin A, Gomez-Baggethun E, and Muradian R. 2014. Social Equity Matters in Payments for Ecosystem Services. Bioscience, October. bioscience.oxfordjournals.org. 10.1093/biosci/biu146.

[CR48] Persha, L., Fischer, H., Chhatre, A., Agrawal A., and Benson, C. (2010). Biodiversity Conservation and Livelihoods in Human-Dominated Landscapes: Forest Commons in South Asia. Biological Conservation 143 (12): 2918–25. Elsevier.

[CR49] Persha, L., Agrawal, A., and Chhatre, A. (2011). Social and Ecological Synergy: Local Rulemaking, Forest Livelihoods, and Biodiversity Conservation. Science 331(6024): 1606–8. science.sciencemag.org. 10.1126/science.1199343 21436453

[CR50] Robbins, P. (1998). Paper Forests: Imagining and Deploying Exogenous Ecologies in Arid India. Geoforum; Journal of Physical, Human, and Regional Geosciences 29 (1): 69–86. Elsevier.

[CR51] Robichaud, W., Marsh C. W., Southammakoth S., and Khounthikoummane S. 2001. Review of the National Protected Area System of Lao PDR. Lao-Swedish Forestry Programme.

[CR52] Salafsky N, Wollenberg E (2000). Linking Livelihoods and Conservation: A Conceptual Framework and Scale for Assessing the Integration of Human Needs and Biodiversity. World Development.

[CR53] Sandbrook, C. G. 2010. Putting Leakage in Its Place: The Significance of Retained Tourism Revenue in the Local Context in Rural Uganda. *Journal of International Development* 22 (1): 124–36. John Wiley & Sons, Ltd.

[CR54] Satyal, P. (2011). Shifting Conceptions of Social (in)justice in Nepal. New Angle: Nepal Journal of Social Science and Public Policy 1(1): 49–64. ueaeprints.uea.ac.uk.

[CR55] Saypanya, S., Hansel, T., Johnson, A., Bianchessi, A., and Sadowsky, B. (2013). Effectiveness of a Social Marketing Strategy, Coupled with Law Enforcement, to Conserve Tigers and Their Prey in Nam Et Phou Louey National Protected Area, Lao People’s Democratic Republic. Conservation Evidence 57–66.

[CR56] Scherr, S., White A., and Kaimowitz D. 2003. A New Agenda for Achieving Forest Conservation and Poverty Alleviation: Making Markets Work for Low-Income Producers. Forest Trends*.* CIFOR, Bogor, Washington.

[CR57] Schlosberg D (2007). Defining Environmental Justice: Theories, Movements, and Nature.

[CR58] Scott JC (1987). Weapons of the Weak : Everyday Forms of Peasant Resistance.

[CR59] Sen A. (1999). The Possibility of Social Choice. The American Economic Review. JSTOR: 349–378.

[CR60] Sikor, T., and Cầm, H. (2016). REDD+ on the Rocks? Conflict over Forest and Politics of Justice in Vietnam. Human Ecology: An Interdisciplinary Journal 44: 217–27. Springer. 10.1007/s10745-016-9821-1 PMC4831992 27122653

[CR61] Stears M, Bell DA, de Shalit A (2003). The Political Conditions of Social Justice. Forms of Justice.

[CR62] Taylor C, Taylor C (1994). The Politics of Recognition. Multiculturalism: Examining the Politics of Recognition.

[CR63] Terborgh J (1999). Requiem for Nature.

[CR64] Tessema, M. E., Lilieholm, R. J., Ashenafi, Z. T., and Leader-Williams, N. (2010). Community Attitudes toward Wildlife and Protected Areas in Ethiopia. Society & Natural Resources 23 (6): 489–506. Taylor & Francis.

[CR65] Thøgersen, J. (2004). A Cognitive Dissonance Interpretation of Consistencies and Inconsistencies in Environmentally Responsible Behavior. Journal of Environmental Psychology 24(1): 93–103. Elsevier.

[CR66] Tumusiime, D. M., and Vedeld, P. (2015). Can Biodiversity Conservation Benefit Local People? Costs and Benefits at a Strict Protected Area in Uganda. Journal of Sustainable Forestry 34(8): 761–86. Taylor & Francis.

[CR67] van der Toorn J, Tyler TR, Jost JT (2011). More than Fair: Outcome Dependence, System Justification, and the Perceived Legitimacy of Authority Figures. Journal of Experimental Social Psychology.

[CR68] Vodouhê, F. G., Coulibaly, O., Adégbidi, A., and Sinsin, B. (2010/9). Community Perception of Biodiversity Conservation within Protected Areas in Benin. Forest Policy and Economics 12 (7): 505–512.

[CR69] Walker, G. (2012). Environmental Justice: Concepts, Evidence and Politics. Routledge.

[CR70] Walpole, M. J., and Goodwin, H. J. (2001). Local Attitudes towards Conservation and Tourism around Komodo National Park, Indonesia. Environmental Conservation 28(02): 160–66. Cambridge University Press.

[CR71] Watts JD, Vihemaki H, Boissière M, Rantala S, Colfer CJP, Pfund JL (2011). Information Flows, Decision-Making and Social Acceptability in Displacement Processes. Collaborative Governance of Landscape Mosaics.

[CR72] Wells M, Guggenheim S, Khan A, Wardojo W, Jepson P (1999). Investing in Biodiversity: A Review of Indonesia’s Integrated Conservation and Development Projects.

